# Key Drivers of Low Covid-19 Vaccine Uptake in Tanzania

**DOI:** 10.48327/mtsi.v3i1.2023.307

**Published:** 2023-01-17

**Authors:** Mohamed Yunus Rafiq, Ibrahim Simiyu, Hannah Wheatley, Bourema Sissoko, Zachary Enumah, Kheri Tungeraza, Brian J. Hall

**Affiliations:** 1 Department of Social Sciences, New York University Shanghai, China; Center for Global Health Equity, New York University Shanghai, China; 2 ResearchCom, Dar es Salaam, Tanzania; 3 CIVICUS, South Africa; 4 Institute of Anthropology, School of Social Development, East China Normal University, Shanghai, China; 5 Johns Hopkins Bloomberg School of Public Health, Baltimore, USA; 6 Muhimbili National Hospital, Dar es Salaam, Tanzania; 7 Center for Global Health Equity, New York University Shanghai, China; Health, Behavior, and Society

**Keywords:** Hésitation vaccinale, Vaccin, Covid-19, Tanzanie, Afrique de l'Est, Vaccine hesitancy, Vaccine, Covid-19, Tanzania, East Africa

## Abstract

L'OMS définit l'hésitation vaccinale comme un retard dans l'acceptation ou le refus des vaccins malgré la disponibilité des services de vaccination. C'est un phénomène complexe qui varie en fonction du temps, du lieu et des vaccins. Dans cette tribune, nous mettons en évidence la variation spécifique contextuelle de la réticence au vaccin Covid-19 en Tanzanie. Nous suggérons que l'hésitation vaccinale contre la Covid-19 en Tanzanie est influencée par une charge élevée de maladies infectieuses, de faibles capacités de test et des caractéristiques démographiques.

## Abstract (en)

WHO defines vaccine hesitancy as delay in acceptance or refusal of vaccines despite the availability of vaccine services. It is a complex phenomenon that varies through time, place and vaccines. In this comment, we highlight the context-specific variation of Covid-19 vaccine hesitancy in Tanzania. We suggest Covid-19 hesitancy in Tanzania is influenced with high burden of infectious disease, poor testing capabilities and demographic characteristics.

## Key Message

- Vaccination rates in Tanzania remain low despite the availability of vaccines.
- Cultural and historical factors effect vaccine uptake but play a minor role.
- Covid-19 hesitancy in Tanzania is influenced by multiple factors including high burden of disease, inadequate testing capabilities and demographic characteristics.

At the onset of the Covid-19 pandemic, Tanzania was globally known as a Covid-19 denier. This changed after the unexpected death of President Magufuli in April 2021. The new President, Samia Suluhu Hassan, immediately formed a Covid-19 taskforce that legitimized public health authorities. Tanzania has since established national vaccine centers, released regular surveillance updates and implemented standard Covid-19 safety measures. Despite these actions, vaccination rates, by the end of 2021, have hovered at 32% (11 million individuals) fully vaccinated, due to vaccine hesitancy driven by high endemic infectious disease burden, inadequate diagnosis, and population demographics [5]. However, with increased community outreach strategies the percentage of fully vaccinated individuals increased to 48% (29 millions) by the end of 2022 [8].

First, the burden of other endemic infectious diseases, including non-Covid-19 respiratory infections, HIV/AIDS, and tuberculosis is already high; additional Covid-19 mortality and morbidity do not significantly motivate vaccination uptake [2]. Tanzania reported only 778 Covid-19 deaths from January 2020 to February 2022 compared to 28,000 HIV/AIDS deaths from April 2020 to May 2021 [4, 5].

Second, access to timely infectious disease testing is lacking. Diagnosis of endemic infectious diseases, including malaria, typhoid, and tuberculosis, remain largely inaccessible and unreliable, and Covid-19 testing is no exception [3]. Covid-19 sample collection is decentralized, but laboratory testing is centralized in one national laboratory in Dar es Salaam. Results are available in 2 to 5 days outside Dar es Salaam, and in Dar es Salaam, it takes 24 to 48 hours. Due to earlier policies, some who tested positive for Covid-19 were given negative results as it was unpopular to state the disease existed in the country, which created an impression of low-risk exposure as people were unaware that loved ones were infected or died of Covid-19 [1].

Although the history and enduring effects of epidemiological and bodily harms caused by European colonial medical and health programs is a factor in how Africans judge current vaccine initiatives, it is unlikely to be a significant factor because Tanzania has one of the highest childhood vaccination rates in the world, and these childhood vaccines also are from Euro-American sources [6]. However, Tanzania's young demographic structure is likely a strong driver of vaccine hesitancy because less than 3% of the population is over 65 years, currently the age group at the highest risk for severe Covid-19. This furthers the perception that Covid-19 is not a significant threat in Tanzania [7].

In Tanzania, high endemic infectious disease burden, poor diagnostic infrastructure, and the young demographic structure of the country drive low Covid-19 vaccine uptake. Current vaccine rates highlight the existing insufficiency in infectious disease management. Public health educational campaigns must continue encouraging widespread vaccination, and government intervention should normalize and improve infectious disease care.

## Message Clé

- Les taux de vaccination en Tanzanie restent faibles malgré la disponibilité des vaccins.
- Les facteurs culturels et historiques affectent l'acceptation de la vaccination mais jouent un rôle mineur.
- La réticence au vaccin contre la Covid-19 en Tanzanie est influencée par de multiples facteurs, notamment une charge de morbidité élevée, des capacités de test inadéquates et des caractéristiques démographiques.

Au début de la pandémie de Covid-19, la Tanzanie était mondialement connue comme déniant la Covid-19. Cela a changé après la mort inattendue du président Magufuli en avril 2021. La nouvelle présidente, Samia Suluhu Hassan, a immédiatement formé un groupe de travail sur la Covid-19 qui a impliqué les autorités de santé publique. La Tanzanie a depuis créé des centres nationaux de vaccination, publié des mises à jour régulières de la surveillance et mis en œuvre des mesures de sécurité standard contre la Covid-19. Malgré ces actions, les taux de vaccination, à la fin de 2021, n'ont pas dépassé 32% (11 millions de personnes entièrement vaccinées), en raison des réticences à se faire vacciner, de la charge élevée des maladies infectieuses endémiques, d'un diagnostic inadéquat et du profil démographique de la population [5]. Cependant, avec l'augmentation des stratégies de sensibilisation communautaire, le pourcentage d'individus entièrement vaccinés a atteint 48% (29 millions) fin 2022 [8].

Premièrement, le fardeau des autres maladies infectieuses endémiques, notamment les infections respiratoires autres que la Covid-19, le VIH/sida et la tuberculose, est déjà élevé; la mortalité et la morbidité supplémentaires liées à la Covid-19 ne motivent pas de manière significative la vaccination [2]. La Tanzanie n'a signalé que 778 décès dus à la Covid-19 de janvier 2020 à février 2022, contre 28 000 décès dus au VIH/sida d'avril 2020 à mai 2021 [4, 5].

Deuxièmement, l'accès aux tests de dépistage des maladies infectieuses en temps opportun fait défaut. Le diagnostic des maladies infectieuses endémiques, notamment le paludisme, la typhoïde et la tuberculose, reste largement inaccessible et peu fiable, et les tests Covid-19 ne font pas exception [3]. La collecte des échantillons Covid-19 est décentralisée, mais la réalisation des tests de laboratoire est centralisée au laboratoire national à Dar es Salaam. Les résultats sont disponibles en 2 à 5 jours en dehors de Dar es Salaam, et à Dar es Salaam, cela prend 24 à 48 heures. En raison de politiques antérieures, certaines personnes ayant été testées positives pour la Covid-19 ont reçu des résultats négatifs car il était impopulaire de déclarer que la maladie existait dans le pays, ce qui a créé une impression d'exposition à faible risque car les gens ne savaient pas que des êtres chers étaient infectés ou décédés de la Covid-19 [1].

Bien que l'histoire et les effets durables des dommages épidémiologiques et corporels causés par les programmes médicaux et sanitaires coloniaux influent sur le jugement des Africains concernant la vaccination, il est peu probable que ce facteur soit significatif car la Tanzanie a l'un des taux de vaccination infantile les plus élevés du monde, alors que ces vaccins infantiles proviennent également du monde occidental [6]. La structure démographique jeune est probablement un puissant moteur de la réticence à la vaccination, car moins de 3% de la population a plus de 65 ans, actuellement le groupe d’âge le plus à risque de Covid-19 grave. Cela renforce la perception que la Covid-19 n'est pas une menace importante en Tanzanie [7].

En Tanzanie, le fardeau élevé des maladies infectieuses endémiques, l'insuffisance des infrastructures de diagnostic et la structure démographique du pays majoritairement composée de jeunes, entraînent un faible recours au vaccin contre la Covid-19. Les taux de vaccination actuels mettent en évidence la médiocre prise en charge des maladies infectieuses. Les campagnes d’éducation pour la santé publique doivent continuer à promouvoir la vaccination à grande échelle, et l'intervention gouvernementale doit normaliser et améliorer les traitements contre les maladies infectieuses.
